# Hearing assessment of infants with congenital syphilis

**DOI:** 10.1590/2317-1782/e20240338en

**Published:** 2026-04-17

**Authors:** Pâmela da Silva Panassol, Luciana Friedrich, Leticia Petersen Schmidt Rosito, Amanda Zanatta Berticelli, Andréa Lucia Corso

**Affiliations:** 1 Programa de Pós-graduação em Saúde da Criança e do Adolescente, Universidade Federal do Rio Grande do Sul – UFRGS - Porto Alegre (RS), Brasil.; 2 Departamento de Pediatria, Universidade Federal do Rio Grande do Sul – UFRGS - Porto Alegre (RS), Brasil.; 3 Serviço de Neonatologia, Hospital de Clínicas de Porto Alegre - Porto Alegre (RS), Brasil.; 4 Departamento de Otorrinolaringologia, Universidade Federal do Rio Grande do Sul – UFRGS - Porto Alegre (RS), Brasil.; 5 Serviço de Otorrinolaringologia, Hospital de Clínicas de Porto Alegre - Porto Alegre (RS), Brasil.

**Keywords:** Hearing Loss, Congenital Syphilis, Auditory Evoked Potentials, Neonatal Hearing Screening, Audiologic Diagnosis

## Abstract

**Purpose:**

To evaluate the presence of hearing loss in the first months of life in infants treated for congenital syphilis at birth.

**Methods:**

Prospective study including all neonates born in a University public hospital in South Brazil, who needed treatment for congenital syphilis after birth in the Neonatal Unit, between 2021 and 2022. Otorhinolaryngologic and audiologic evaluations were performed at a mean age of 3.3 months, concomitantly with the first post-discharge appointment in the congenital syphilis outpatient clinic, through frequency-specific auditory brainstem response.

**Results:**

Sixty-five patients were included in the study. All subjects underwent Neonatal Hearing Screening before being discharged from the hospital, consisting of transient evoked otoacoustic emission and automated auditory brainstem response tests. Sixty-one patients (93.8%) passed both screening tests and four (6.2%) failed both tests. Concerning the 4 newborns who failed, 2 returned for retesting and had normal results, 1 patient did not show up for retesting, and 1 patient failed both retests. Follow-up was concluded by 23 infants (35.4% of the initial sample) who underwent Frequency-Specific Auditory Brainstem Response testing. The other patients did not attend to the appointments. Regarding the 23 patients that concluded the follow-up, no interaural response asymmetries suggestive of retrocochlear involvement were observed. Additionally, no changes were found in the absolute latencies of waves I, III, and V, or in the interpeak intervals I–III, III–V, and I–V. The Frequency-Specific Auditory Brainstem Response evaluation did not present any abnormalities in the electrophysiological thresholds at the tested frequencies.

**Conclusion:**

No signs of hearing loss were found in patients treated for congenital syphilis during neonatal period, either in the Neonatal Hearing Screening or in the Frequency-Specific Auditory Brainstem Response during follow-up.

## INTRODUCTION

Sexually transmitted infections represent a public health challenge due to their economic, medical and social consequences. When affecting pregnant women, many of these pathologies can result in congenital infections, which can be related to neurodevelopmental delay, visual and hearing impairment. Among congenital infections, syphilis is of great epidemiological importance because, despite accessible, effective, and efficient treatment, there are still high incidence rates mainly in middle and low-income countries, with low treatment rates at public health centers and, as a result, a high prevalence of congenital syphilis (CS)^(1,2)^. In Brazil, although prenatal care and treatment with penicillin are provided free of charge through the Unified Health System (SUS), the country still shows low rates of adequate treatment for gestational syphilis. According to the Ministry of Health’s 2023 Epidemiological Bulletin, in 2022 only 82.6 % of pregnant women diagnosed with syphilis received proper treatment^(3)^.

In the last decade, there has been a progressive increase in the incidence of syphilis in pregnant women. In 2022, Brazil recorded a detection rate of 32.4 cases of gestational syphilis per 1,000 live births (LBs), an increase of 15.5% over the previous year. The detection rate in the southern region of Brazil (33.8 cases/1,000 LBs) is higher than the national average^(3)^.

The state of Rio Grande do Sul also has the highest CS rates, and among the state capitals, Porto Alegre has the highest incidence rate of the disease in Brazil (39.4 cases/1,000 LBs)^(3)^.

Syphilis is a systemic bacterial infection caused by *Treponema pallidum* that can be transmitted via sexual intercourse, blood transfusion, or vertically, when transmitted from pregnant women to her fetus, through transplacental route, mostly in the 3rd trimester. Syphilis during pregnancy must be treated with intramuscular Benzilpenicilline 2.400.000 UI, once a week, for 3 consecutive weeks, in order to reduce the risk of fetal infection. Non-treponemal tests should be taken monthly to confirm titer reduction and if re-infection occurs, a new complete treatment should be taken.

If fetal infection occurs, miscarriage, fetal hydrops, premature birth and even fetal death / stillbirth can take place, mainly in 1st trimester gestational infections. Infected newborns (NB) are mostly asymptomatic (80%), or can present with lymphadenomegaly, hepatosplenomegaly, maculo-papular rash, skin lesions, jaundice, nasal discharge, hemolytic anemia, leucopenia and thrombocytopenia, respiratory distress, skeletal abnormalities or even neurologic problems due to central nervous system (CNS) involvement. Even asymptomatic newborns, if not properly treated, can develop signs of congenital syphilis between 1 and 3 months of age. If infected newborns are not treated properly in the first months of life, important consequences such as bone, cognitive, neurological, visual and auditory sequelae can occur^(2,4-6)^.

Neonates and infants with a history of congenital infection are known to be at risk for hearing loss (HL). The incidence of sensorineural HL in CS can vary from 25 to 38% and the onset can be sudden or progressive, unilateral or bilateral. In addition, CS can lead to HL later in life, around 2 years of age. However, studies in the literature relating the disease to HL are scarce and insufficiently explored. In addition, the prevalence of sensorineural HL in childhood caused by CS is still not well defined^(7-11)^.

Early detection of this loss is important for early intervention. This study aims to assess the presence of HL in the first months of life in infants treated for CS in the neonatal period. Understanding these responses will help to develop an audiologic assessment protocol for hearing monitoring in these populations.

## METHODS

**Figure 1:**
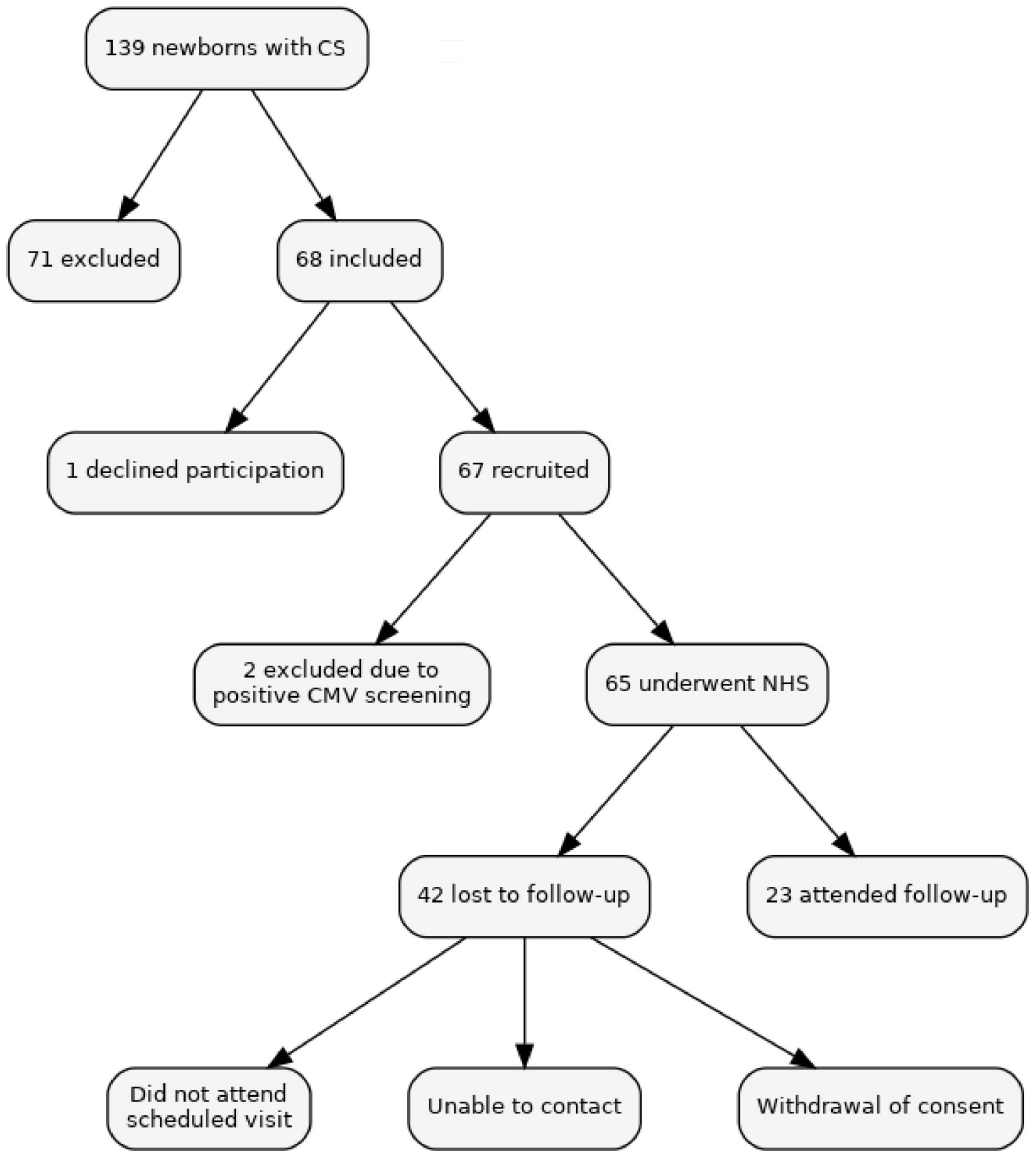
Sample selection and monitoring

A prospective study was conducted in a tertiary hospital in southern Brazil. The sample consisted of infants diagnosed with CS and treated in the neonatal period, born between May 2021 and December 2022, and subsequently followed up at the hospital's CS outpatient clinic. Figure 1 shows the flowchart describing the sample selection and monitoring.

Diagnoses of gestational syphilis and CS were made according to the criteria used by the Brazilian Ministry of Health (MoH), as well as the criteria for considering a pregnant woman to be adequately treated^(12)^.

Following the Brazilian Ministry of Health (MoH) guidelines, pregnant women must be submitted to treponemal Rapid Tests (RT) in each of the 3 trimesters of pregnancy. The sensitivity and specificity of rapid tests for syphilis are high, ranging from 90 to 100%^(12,13)^. If a positive RT occurs, treatment should be prescribed and a non-treponemal test (VDRL = Venereal Disease Research Laboratory) should be taken monthly to follow-up maternal infection. When arriving at the Obstetric Centre, these patients collect a new VDRL test, to be compared to the NB titers as soon after birth. Pregnant women who had not received prenatal care or any testing for syphilis during antenatal care were submitted to the RT as soon as they arrived at the maternity center. If positive, VDRL was performed also to compare to NB’s titration.

All neonates born from mothers with gestational syphilis follow the MoH guidelines for specific evaluation (hematological and cerebrospinal fluid analysis and long bones X-rays) and, if CS is diagnosed, they are admitted to the Neonatology Unit (NU) to a 10-day intravenous Crystalline Penicillin treatment.

All NB underwent neonatal hearing screening (NHS) prior to hospital discharge, following the institution’s standard protocol. The screening included both electroacoustic and electrophysiological assessments: transient evoked otoacoustic emissions (TEOAE) followed by automated auditory brainstem response (AABR), performed simultaneously. In cases of failure in either or both tests, a retesting was scheduled within 15 days on an outpatient basis, using the same procedures. If there is a failure in the NHS retesting, patient is immediately forwarded to audiologic diagnoses through otorhinolaryngologic and audiologic assessments, conducted in the otorhinolaryngology and speech and hearing departments of the same hospital.

Exclusion criteria of this study included coexisting congenital infections (rubella, toxoplasmosis, cytomegalovirus [CMV] and human immunodeficiency virus [HIV]), major congenital malformations or genetic syndromes that could lead to HL. Also were excluded preterm neonates under 34 weeks of gestational age, patients with perinatal asphyxia, hyperbilirubinemia with levels indicative of exsanguineous transfusion and those who had used ototoxic medications in the neonatal period (furosemide, vancomycin or aminoglycosides for more than 5 days). These exclusion criteria aimed to reduce the chance of diagnosing HL caused by these risk factors and wrongly attribute it to CS.

Patients' medical records were analyzed to identify demographic data related to pregnancy, gestational syphilis and its treatment, delivery, the NB, tests related to CS and its treatment, as well as other complications during the perinatal period. Urine was collected to test for CMV by polymerase chain reaction (PCR) during hospitalization. This test was performed to prevent possible CMV infections from being mistakenly attributed to CS.

Patients were forwarded to the otorhinolaryngologic and audiologic evaluations with 3 months of age, concomitantly with the first follow-up appointment at the CS outpatient clinic, in the same Hospital. The scheduling was planned for this period in order to prevent patient dropout and withdrawal by their caregivers in case of needing to come to the hospital more than once.

In the follow-up audiologic assessment, we were not able to perform acoustic immittance measurements to assess middle ear status due to the unavailability of the necessary equipment at the facility during the study period. Nevertheless, the evaluation of the external auditory canal and tympanic membrane was conducted through otorhinolaryngological inspection using a hand-held otoscope by an experienced pediatric otorhinolaryngologist. Although immittance testing provides objective data on middle ear function, the clinical otoscopic examination allowed for the identification of visible abnormalities or signs of middle ear pathology, ensuring a preliminary assessment of the ear's condition in the absence of instrumental measures. The pediatric otorhinolaryngologist also took patients’ medical history focusing on hearing issues.

The auditory brainstem response (ABR) evaluation comprised two distinct protocols, each designed to address specific diagnostic objectives. The click-evoked ABR was employed to assess the functional integrity of the auditory pathways within the brainstem. In contrast, the frequency-specific ABR (FS-ABR) was performed to estimate electrophysiological hearing thresholds across specific frequencies, providing frequency-specific information about auditory sensitivity.

To perform the ABR, the skin was first cleaned with Nuprep® abrasive paste. Disposable Meditrace Kendall™ pediatric electrodes were then placed at the following locations: near the scalp, the active electrode (Fz); on the forehead, the ground electrode (Fpz); and on the left (M1) and right (M2) mastoids. EarTone 3A earphones were used. An Interacoustics Eclipse EP25 was used. The impedance of the electrodes was checked prior to the start of the test – the evaluation was only started if the impedance was less than or equal to 5Ω (ohms). During the test, the infants remained in natural sleep on their mother's or caregiver's lap.

The auditory pathway integrity test was performed using the ABR with a click stimulus at an intensity of 80 dBHL, with 2000 stimuli presented monaurally. Two samples were taken in the rarefied polarity and one in the condensed polarity to confirm the responses. The absolute latencies of waves I, III and V, the interpeak latencies I-III, III-V and I-V, and the interaural difference in wave V latency were analyzed.

After completion of the integrity test, electrophysiologic threshold testing was begun at 500, 1000, 2000 and 4000 Hz using Narrow Band CE-Chirp® stimulation and alternating polarity. The electrophysiologic threshold was considered to be the lowest value at which wave V was present and was observed in at least two samples to ensure reproducibility. Thresholds were measured in the following order: 2000, 500, 4000 and 1000 Hz, in ascending order, and were considered normal when a response was found at 30, 35, 25 and 30 dB, respectively.

The experience of two evaluators was used to mark the waves and define the thresholds, and they were considered valid only if there was agreement between the markings.

The data were analyzed using the Statistical Package for Social Science (SPSS 20.0). Quantitative variables were described using means and standard deviations, and qualitative variables were described using frequencies and percentages.

All mothers or guardians signed an informed consent form. The research was approved by the institutional ethics committee (Approval number: 5.724.613).

## RESULTS

Between May 2021 and December 2022, 4,565 LBs were delivered at the institution. A total of 139 newborns with a diagnosis of CS were initially recruited, resulting in a prevalence of newborns with CS of 30.4 per 1,000 LBs.

Of the patients born with CS, 68 met the inclusion and exclusion criteria and constituted the study sample. Of these, one family member/guardian refused to participate in the study. Thus, the initial sample consisted of 67 patients. The characterization of the sample is shown in Table 1.

**Table 1:** Demographic data of the initial sample

Variables	*n* = 67
Maternal age (years)*	24.60 ± 5.14
Number of PN consultations*	7.24 ± 3.77
GA (weeks)*	39.26 ± 1.41
Sex	
Female	27 (40.3%)
Male	40 (59.7%)
NB age (months)^*^	3.30 ± 0.98
NB classification	
AGA	47 (70.1%)
SGA	13 (19.4%)
LGA	7 (10.4%)
CMV	
Not detected	40 (59.7%)
Detected	2 (3.0%)
Not performed	25 (37.3%)

* mean ± standard deviation

Caption: PN = prenatal; GA = gestational age; NB = newborn; AGA = appropriate for gestational age; SGA = small for gestational age; LGA = large for gestational age; CMV = cytomegalovirus

Regarding CS, all NBs were clinically asymptomatic. No patients were excluded due to prematurity, concomitant congenital infections, congenital anomalies or genetic syndromes, perinatal asphyxia or ototoxic drugs. The results of the examination tests are shown in Table 2.

**Table 2:** Tests for congenital syphilis (*n* = 67)

	Normal	Altered
**Long bone X-ray**	66 (98.5%)	1 (1.5%)
**Cerebrospinal fluid**	66 (98.5%)	1 (1.5%)
**Blood count**	67 (100%)	0 (0%)

A total of 42 urine samples were collected for CMV analysis (62.7% of the total). Only 2 of them showed a positive result in the PCR test and therefore were excluded from the study. The prevalence of congenital CMV in the sample was 4.76%. It was not possible to collect a urine sample of all included NB due to neonate discharge before researchers could ask the mother to sign the informed consent and authorize to collect a urine sample of the NB.

The 65 individuals who made up the final sample underwent the NHS before being discharged from the hospital. Sixty-one patients (93.8%) had normal results on both tests and 4 (6.1%) failed both tests. Of the 4 failures, 2 had normal re-tests, 1 failed to show up for the testing and 1 had a re-test with a unilaterally altered AABR and was forwarded to specific audiologic diagnosis.

During follow-up, 23 out of 65 patients (35.4% of the sample) attended the audiologic assessment, which included both click-evoked auditory brainstem response (ABR) and frequency-specific ABR (FS-ABR). The evaluations were conducted at a mean age of 3.3 months. The remaining patients did not attend the scheduled follow-up appointment and, therefore, could not be evaluated or included in this phase of the study.

No abnormalities were observed between ears in the click-evoked ABR that would suggest retrocochlear involvement. The parameters assessed in this test showed no changes in the values of the absolute latencies of waves I, III and V and the interpeak intervals I-III, III-V and I-V in both ears (Table 3).

**Table 3:** Absolute latency values, interpeak intervals and interaural difference

Variables	*n* = 23
Right ear - mean ± standard deviation	
Wave I (ms)	1.48 ± 0.27
Wave III (ms)	4.12 ± 0.35
Wave V (ms)	6.20 ± 0.46
Left ear - mean ± standard deviation	
Wave I (ms)	1.54 ± 0.46
Wave III (ms)	4.10 ± 0.41
Wave V (ms)	6.17 ± 0.45
Right ear - mean ± standard deviation	
Interpeak I-III (ms)	2.65 ± 0.16
Interpeak III-V (ms)	2.08 ± 0.26
Interpeak I-V (ms)	4.72 ± 0.36
Left ear - mean ± standard deviation	
Interpeak I-III (ms)	2.56 ± 0.23
Interpeak III-V (ms)	2.07 ± 0.29
Interpeak I-V (ms)	4.63 ± 0.25
Interaural difference - mean ± standard deviation	0.03 ± 0.21

Caption: ms = milliseconds

No changes in electrophysiologic thresholds were observed in the diagnostic ABR at the specific frequencies studied.

Regarding the patients who failed the NHS, the first patient did not return for the retest, but underwent the study follow-up at 3 months, with hearing integrity values and electrophysiologic thresholds within the normal range.

The other patient had an abnormal NHS test and retest and did not attend the follow-up of this study. However, after analysis of the medical records, it was found that he had been followed up according to the NHS protocol at the hospital's Auditory Rehabilitation Service, where diagnostic testing was performed. According to the protocol of the service, a hearing integrity test was performed (a delay in the block of waves I, III and V on the right side was found) and electrophysiologic thresholds were investigated using the steady-state auditory evoked potential (an altered air conduction and normal bone conduction on the right side were observed). It was concluded that the patient had tests compatible with conductive HL on the right and normal tests on the left.

## DISCUSSION

The results of this study provide important insights into the impact of CS on the auditory system, being possibly the first to evaluate HL in these patients through both TEOAE and ABR in the neonatal period, and also testing these patients with 3 months of age using methods that assess both the integrity of the auditory pathway and frequency-specific electrophysiological thresholds. All NB evaluated in this study presented results within normal ranges, consistent with the majority of studies that assessed this group through NHS in order to investigate HL^(14-19)^.

The electrophysiological evaluation conducted during the follow-up of patients at 3 months of age using click-evoked ABR, aimed at assessing the integrity of the auditory pathway, did not show any response asymmetry between the ears that would suggest retrocochlear involvement. Additionally, no abnormalities were found in the absolute latency values or the interpeak intervals for both ears. The only studies found in the literature using the same tests were performed solely in the neonatal period and did not include the investigation of frequency-specific electrophysiological thresholds^(20,21)^.

In a North American study conducted by Ghogomu et al.^(22)^, which aimed to compare the epidemiology of unilateral pediatric sensorineural hearing loss before and after the implementation of newborn hearing screening, no association was found between cases of congenital syphilis and this type of hearing loss.

In order to gain more information about the functioning of outer hair cells (OHC), Ribeiro et al.^(20)^ evaluated the audiological findings of newborns exposed to adequately treated maternal syphilis during pregnancy in a public hospital, comparing response amplitudes between individuals exposed to maternal syphilis and those without risk indicators for auditory disorders (RIFHL). The group exposed to maternal syphilis showed lower response amplitudes at all frequencies analyzed. These findings may indicate a cochlear pathology; however, there is not yet enough data to explain its clinical significance.

The practical implications of the results of this study should be considered, as it presents novel findings regarding the investigation of frequency-specific electrophysiological thresholds in the first months of life. No studies using the same evaluation method in this age group were found in the literature. Therefore, further studies are needed to conduct complementary analyses of the electrophysiological parameters in this population.

Even though no sensorineural hearing loss was found in the studied population during the first months of life, it remains important to monitor neonates treated for congenital syphilis, as congenital infections can present with late-onset hearing loss manifestations^(3,23,24)^. It is essential that these patients receive both medical and audiological follow-ups at least once a year until the age of two, again in preschool, and during school age when the process of learning to read and write begins. Additionally, follow-up should be conducted whenever there is a suspicion of hearing loss by parents and/or caregivers, regardless of the child's age. However, it is worth noting a study conducted at an Italian children's hospital, which monitored the hearing of 92 children with congenital infections up to the age of four, and found no cases of late-onset hearing loss related to congenital syphilis.

Another distinguishing feature of this study is the investigation of CMV in urine, which is not part of routine clinical practice. This coinfection was not examined in any of the few studies that described HL in patients with CS^(25,26)^. In the present study, the prevalence of congenital CMV coinfection was 4.76% in the sample of NB treated for CS. This prevalence is significantly higher than the 1% reported in the literature for newborns without other risk factors^(27,28)^. It is valuable to note that, although it was not possible to test all 67 initially included NB, a 4.76% prevalence of congenital CMV (2/46 NB) would result at most in 3 or 4 positive tests in all the 65 included NB.

However, this study is not without limitations. Of the total patients recruited, only 34.3% attended the follow-up appointment at the congenital syphilis clinic at 3 months of age, when an otolaryngological and audiological evaluation was also scheduled. The high dropout rate was a significant challenge for the researchers. The literature describes a high rate of non-attendance to follow-up care in patients treated for congenital syphilis, particularly in developing countries like Brazil. Contributing factors include socioeconomic disadvantages, difficulties in accessing and mobility to healthcare services, the stigma associated with sexually transmitted infections, and the misleading belief among parents and caregivers that syphilis, often asymptomatic in both the pregnant woman and the newborn, does not lead to long-term consequences or complications, making follow-up seem unnecessary and futile^(29-31)^. Additionally, it is important to note that all evaluated patients were asymptomatic for congenital syphilis at birth. No studies were found investigating auditory changes in infants with symptomatic congenital syphilis at birth.

This study contributes to questioning the need to maintain CS as a high-risk disease, particularly in asymptomatic NB. However, given the possibility of late manifestations, continued follow-up is essential. It is also crucial to understand the reasons behind the high percentage of follow-up dropout in this patient group so that strategies can be developed to promote more effective participation of these families in follow-up programs for newborns treated for congenital syphilis during the neonatal period. Further studies with larger numbers of patients and a longer follow-up are needed to confirm these findings and to reassign CS to the classic high-risk factor for HL group.

## CONCLUSIONS

In this study, no sensorineural HL was identified in children with CS. The evaluated patients showed no signs of HL detected by NHS or at the 3-month follow-up assessment of auditory pathway integrity and electrophysiological thresholds.
